# Chi-miR-4110 promotes granulosa cell apoptosis by targeting Sma- and Mad-related protein 2 (Smad2) in the caprine ovary

**DOI:** 10.1371/journal.pone.0181162

**Published:** 2017-07-13

**Authors:** Xiaopeng An, Yuxuan Song, Jinxing Hou, Yue Zhang, Kaiwen Chen, Haidong Ma, Xinyan Zhao, Guang Li, Kexin Gao, Shan Wang, Binyun Cao, Yueyu Bai

**Affiliations:** 1 College of Animal Science and Technology, Northwest A&F University, Yangling, Shaanxi, P.R. China; 2 Animal Engineering Branch, Yangling Vocational & Technical College, Yangling, Shaanxi, P.R. China; 3 Northwest A&F University of Hospital, Northwest A&F University, Yangling, Shaanxi, P.R. China; 4 Animal Health Supervision Institute of Henan Province, Zhengzhou, Henan, P.R. China; Universitatsklinikum Leipzig, GERMANY

## Abstract

Follicular atresia mainly results from the apoptosis of granulosa cells (GCs). Whilst our previous investigations examined the role of chi-miR-4110 in regulating ovarian function, the present study detected the role of chi-miR-4110 in GC development. We transfected caprine GCs cultured *in vitro* with chi-miR-4110 mimics. Results revealed that chi-miR-4110 decreased mRNA and protein levels of Smad2 by targeting its 3′-untranslated region (3′UTR). FoxC1 and Sp1 mRNA and protein levels markedly increased, whereas those of bHLHe22 significantly decreased (*P*<0.01 or 0.05) in GCs transfected with the chi-miR-4110 mimics. Further studies revealed a significantly higher number of apoptotic cells in GCs transfected with the chi-miR-4110 mimics (*P*< 0.05) than in GCs transfected with mimics negative control. GCs transfected with the chi-miR-4110 mimics exhibited significantly increased mRNA and protein levels of the pro-apoptotic gene *Bax* (*P*<0.01) and significantly decreased expression levels of the anti-apoptotic gene *BCL-2* (*P*<0.01). Smad2 interference (Si-1282) results were consistent with those of the chi-miR-4110 mimics. Previous reports and our results showed that chi-miR-4110 increases Sp1 expression by repressing Smad2. The increase in Sp1 induces p53-upregulated modulator of apoptosis, which increases the relative abundance of Bax and causes caprine GC apoptosis. Our findings may provide relevant data for the investigation of miRNA-mediated regulation of ovarian functions.

## Introduction

Proliferation of ovarian granulosa cells (GCs) is essential in follicular development, maturation, atresia, ovulation and luteinisation [1]. Folliculogenesis is a complex process during which oocytes increase in size and develop into a mature form, accompanied by the proliferation and differentiation of the surrounding GCs [2]. This process is regulated by various extra- and intra-ovarian factors, such as Anti-mullerian hormone (AMH), Smads, Activins, Inhibins, Bone morphogenetic proteins (BMP) and Growth differentiation factor (GDF9) [3, 4].

Smad proteins (Smad1, Smad2, Smad4, Smad3, Smad5 and Smad8) are the central mediators of the transforming growth factor beta (TGFβ) signalling pathway [5]. Previous studies found that TGFβ superfamily ligands and their receptors are expressed in oocytes, GCs and theca cells of ovarian follicles, functioning as intra-ovarian regulatory molecules involved in follicle recruitment, GC and theca cell proliferation/atresia, steroidogenesis, oocyte maturation and luteinisation [6, 7]. Members of the TGFβ family ligands bind to types I and II serine/threonine kinase receptors to initiate signalling by activating Smads proteins, which are intracellular TGFβ signalling mediators [8]. Receptor-regulated Smads (R-Smads) are Smad molecules phosphorylated by type I receptors [9, 10]. Smad2 and Smad3 are activated by GDF-9 and other TGFβ ligands [11]. Smad1, Smad5 and Smad8 are BMP-specific R-Smads. Upon activation, R-Smads interact with the common Smad (Co-Smad4), which acts as a common partner for all R-Smads [12]. Activated R-Smads form oligomeric complexes with Co-Smad4 and translocate into the nucleus, where they regulate target gene expression via interaction with a multitude of other transcription factors (FoxC1, bHLHe22 and Sp1), co-activators and co-repressor [8, 13, 14].

MicroRNAs (miRNAs) regulate gene expression by targeting mRNAs for degradation or blocking mRNA translation by binding to the 3′-untranslated region (3′-UTR) [15]. Within the past decade, miRNAs have been recognised as important regulators in many biological and cellular processes, such as cell proliferation, differentiation, apoptosis and tumorigenesis [16–18]. Although miRNA functions have not been fully elucidated, emerging evidence shows that miRNAs are involved in GC proliferation, ovarian estradiol synthesis and oocyte maturation [19, 20]. The regulatory mechanisms of chi-miR-4110 in GC apoptosis during follicle atresia have not been elucidated. Based on previous findings [21], we hypothesise that chi-miR-4110 has important roles in Smad2 expression and goat ovarian GC survival. To test this hypothesis, we investigated the influence of chi-miR-4110 on apoptosis-related gene expression, follicular GC proliferation, apoptosis and atresia in the goat ovary. Our results may help explain the potential role of chi-miR-4110 in GC apoptosis.

## Materials and methods

### Ethics statement

All animals were maintained in accordance with proclamation No. 5 of the Ministry of Agriculture, China. Sample collection was approved by the Institutional Animal Care and Use Ethics Committee of Northwest A&F University and performed in accordance with the ‘‘Guidelines for Experimental Animals” of the Ministry of Science and Technology (Beijing, China). The Institutional Animal Care and Use Ethics Committee of Northwest A&F University approved this study.

### Goat GC collection and culture

Goat GCs were collected from the ovaries of Guanzhong dairy goats with the previously described follicle isolation method [22, 23]. The cells were seeded in 12 well culture plates at a density of 5 × 10^5^/well in 1 mL of DMEM/F12 with 10% FBS, 100 IU/mL penicillin, and 50 mg/mL streptomycin. The cells were cultured at 37°C in a 5% CO_2_ atmosphere. After 24 h, the cells were washed twice with PBS and changed with fresh medium (DMEM/F12 with 10 mM HEPES, 20 mM L-glutamine, 100 IU/mL penicillin, 50 mg/mL streptomycin, 0.1% BSA, 10 μg/mL transferrin, 4 ng/mL sodium selenite and 10 ng/mL insulin) for 12 h. The cells were then treated with specific reagents for the interval indicated in the text. At the end of each culture period, cells were collected for total RNA and protein isolation.

### RNA isolation, reverse transcription and real-time PCR

Total RNA were extracted from GCs with RNAiso Plus (TaKaRa, Dalian, China) following the manufacturer's instructions. RNA concentration and purity were determined by measuring optical density (OD) at wavelengths of 260 and 280 nm with an Epoch microplate spectrophotometer (BioTek Instruments, Inc., USA). The OD_260/280_ ratios were >1.8 and <2.1 for all samples. The reverse transcription reaction system are showed as follows: (1)Mixed 800 ng of total RNA, 2 μL of 5 × gDNA Eraser Buffer, 1μL of gDNA Eraser and RNase-Free dH_2_O to a final volume of 10 uL, and then incubated the mixture at 42°C for 2 min. (2) added 4 μL of 5 × Prime Script Buffer 2, 1 μL of PrimeScript RT Enzyme Mix 1, 1 μL of RT Primer Mix and RNase-Free dH_2_O to a final volume of 20 uL, and then incubated the mixture at 42°C for 15 min followed by 85°C for 5 S using PrimeScrip RT reagent Kit (TaKaRa, Dalian, China). The cDNA products were stored at -20 ^o^C until further analysis.

Real-time PCR was then performed with a 25 μL reaction volume containing 12.5 μL of SYBR Premix Ex Taq II (TaKaRa, Dalian, China), 2 μL of template cDNA and 1 μM of primers by the CFX Connect Real-Time PCR Detection System (Bio-Rad, CA, USA). Thermal cycling conditions were 95°C for 10 min, followed by 40 cycles at 94°C for 15 s, 60°C for 30 s and 72°C for 30 s. Primers are shown in Table 1. The *GAPDH* gene was used for normalisation. Each experiment was independently repeated at least three times, and the fold change in the expression of each gene was analysed via the 2^-ΔΔCt^ method.

**Table 1:** Sequence information of real-time PCR and Si-907, Si-1151, Si-1282 and Si-NC.

Name	Primer sequence (5'-3')	Tm (°C)
*Smad2*	F: ATGTCGTCCATCTTGCCATT	58
	R: TCCATTCTGCTCTCCTCCAC	
*Sp1*	F: ACAAACGCACACACACAGGT	59
	R: GGGCCTCCCTTCTTATTCTTG	
*FoxC1*	F: CCTGAACGGCATCTACGAGT	59
	R: GGAACCTTGACGAAGCAGTC	
*bHLHe22*	F: GCTGGTTGCCCTTTCTA	58
	R: TCACCATCCAACCTGACAAA	
*Bcl-2*	F: ATGTGTGTGGAGAGCGTCAA	58
	R: GAGACGGCCAGGAGAAATCAA	
*Bax*	F: ATCTCCAATGCGCTTCAGAC	57
	R: TTTGCTTCAGGGTTTCATCC	
*GAPDH*	F: TTCCGCGGCACAGTCAAG	58
	R: TACTCAGCACCAGCATCACC	
Si-907	Sense: GCCUAGGUUUACUCUCCAATT	
	Antisense: UUGGAGAGUAAACCUAGGCTT	
Si-1151	Sense: GCUGGCUCAGUCCGUUAAUTT	
	Antisense: AUUAACGGACUGAGCCAGCTT	
Si-1282	Sense: GGAUUGAACUUCAUCUGAATT	
	Antisense: UUCAGAUGAAGUUCAAUCCTT	
Si-NC	Sense: UUCUCCGAACGUGUCACGUTT	
	Antisense: ACGUGACACGUUCGGAGAATT	

### Target gene prediction of chi-miR-4110

Chi-miR-4110 (5′-UAGCAGCACAGAAAUGUUGGUA-3′) target genes and miRNA binding sites were predicted by miRBase (http://www.mirbase.org/), TargetScan (http://www.targetscan.org/), PicTar (http://pictar.mdc-berlin.de), miRanda (http://www.microrna.org/microrna/home.do) and RNAhybrid (http://bibiserv.techfak.uni-bielefeld.de/rnahybrid/). MiRNA binding sites were predicted according to the minimum free energy hybridisation of RNA sequences and miRNAs (ΔΔG value). The lower (more negative) the value of the energetic score ΔΔG, the stronger the expected binding of the miRNAs to the given site. The combined ΔΔG values of these approaches were considered to reduce possible false positives.

### Plasmid construction and luciferase reporter gene assay

Fragments of *Smad2* 3′UTR containing the predicted chi-miR-4110 binding site were cloned with the following primers: F: 5′-CCG***CTCGAG***GATGTCTTGTTGGGCATAA-3′ and R: 5′-TT***GCGGCCGC***GTGGGAATAACAGATTAGGT-3′. Italicized and bolded letters indicate *Xho*I and *Not*I endonuclease restriction sites. Fragments of *Smad2* 3′UTR were then linked to a pMD19-T vector with a TA Cloning Kit (Invitrogen, CA, USA). The recombinant pMD19-T vectors were digested by *Xho*I and *Not*I endonucleases. Finally, the digested products were inserted between the *Renilla* and firefly luciferase genes in a psiCHECK-2 vector (Promega, WI, USA). The psiCHECK-2 luciferase vector containing the mutant chi-miR-4110 binding site GTATCACAGTATTG***ATGCA***T***G***T (italicized and bolded letters indicate mutated nucleotides), was obtained from Generay Biotech (Shanghai, China). GCs were co-transfected with *Smad2* 3′UTR or its mutant reporter construct, together with the chi-miR-4110 mimics/mimics negative control (NC). The chi-miR-4110 mimics and mimics NC were chemically synthesised and purified by Shanghai GenePharma (Shanghai, China). The chi-miR-4110 mimics and mimics NC were transfected with Lipofectamine 2000 (Invitrogen, Shanghai, China) following the manufacturer’s instructions at a final concentration of 60 nmol/L. Cell lysates were harvested by direct lysis after 36 h of culture. Luciferase activity was measured in triplicate by the Dual Luciferase Assay System (Promega, Madison, USA). *Renilla* luciferase activity was normalised to firefly luciferase activity. Each experiment was independently repeated at least four times.

### Annexin V-FITC assay

GCs transfected with chi-miR-4110 mimics, mimics NC, chi-miR-4110 inhibitor, inhibitor NC, Smad2 siRNAs (Si-907, Si-1151 and Si-1282) or siRNA negative control (Si-NC) were harvested 48 h after transfection. In transfected GCs, the final concentration of siRNAs is 40 nmol/L. The chi-miR-4110 inhibitor is the reverse complementary sequence of chi-miR-4110 mimics, which can be competitive binding to mature chi-miR-4110 sequence and reduce the gene silencing effect of endogenous chi-miR-4110. Table 1 shows the Si-907, Si-1151, Si-1282 and Si-NC sequences. The apoptotic effect was measured by the Annexin V-FITC Apoptosis Detection Kit (KeyGEN, Nanjing, China). Apoptotic cells were quantified by flow cytometry 1 h after cell population staining with Annexin V-FITC and propidium iodide (PI) according to the manufacturer’s instructions.

### Western blotting

GCs were harvested, rinsed twice with PBS, lysed in denaturing lysis buffer containing protease inhibitors (RIPA, Applygen, Beijing, China) for 30 min on ice and then centrifuged (12000 ×*g*) for 15 min at 4°C. Protein concentration in the lysate was determined by a BCA protein assay kit (Vazyme Biotech, Nanjing, China). Exactly 30 μg of protein was separated on a 12% SDS–PAGE gel and transferred to a PVDF membrane (Merck Millipore). The membrane was blocked with 5% non-fat dried milk in Tris-buffered saline containing 0.1% Tween 20 (TBST, pH 7.6) for 1 h at room temperature. The membranes were subsequently incubated overnight at 4°C with a rabbit anti-Smad2 polyclonal antibody (1:200, Bioss, Beijing, China), a rabbit anti-FoxC1 polyclonal antibody (1:200, Bioss, Beijing, China), a rabbit anti-Sp1 polyclonal antibody (1:200, Boster, Wuhan, China), a rabbit anti-bHLHe22 polyclonal antibody (1:500, Proteintech, Beijing, China) or a mouse anti-actin monoclonal primary antibody (1:1000, Beyotime Biotechnology, Jiangsu, China). The blot was then incubated with a horseradish peroxidase (HRP)-conjugated secondary antibody for 1 h at room temperature. Proteins were detected using enhanced chemiluminescence (Advansta, California, USA). Quantification was performed using the Quantity One program (Bio-Rad, California, USA).

### Statistical analysis

All data were presented as mean ± standard deviation. Results were analysed by one- or two-way analysis of variance, followed by a least significant difference Tukey’s test. *P* < 0.05 was considered statistically significant. All statistical analyses were performed by SPSS 16.0.

## Results

### Chi-miR-4110 directly targets the 3′UTR of *Smad2* mRNA

**Fig 1:**
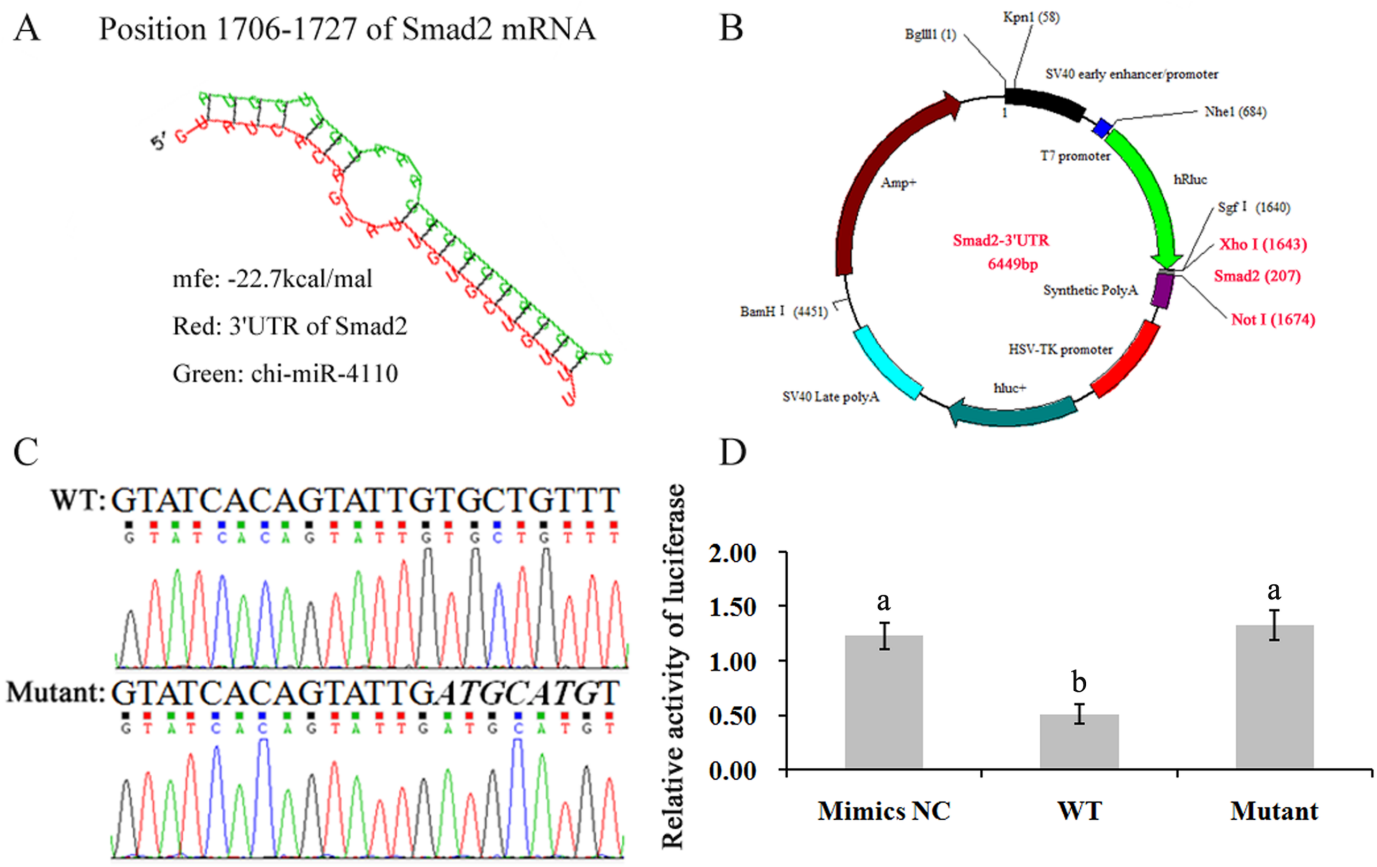
Identification of the chi-miR-4110 target gene. *Smad2* gene was predicted as an important target. (A) chi-miR-4110 binds at positions 1706–1727 of *Smad2* mRNA. (B) The wild-type psiCHECK-2–Smad2–3′UTR (WT) vector and mutant psiCHECK-2–Smad2–3′UTR (Mutant) vector, which contained seven continuous mutant nucleotides, were constructed by a dual luciferase reporter system. (C) Smad2–3′UTR–WT and Mutant sequences. Mutated bases are in italics. (D) The vectors were co-transfected with either chi-miR-4110 mimics or mimics negative control (NC). After 36 h of transfection, GCs were collected and subjected to dual-luciferase assay. Relative luciferase activity was determined by the ratio of firefly to *Renilla* luciferase activity. Data are presented as mean activities ± standard deviation. Different small letters represent a significant difference at the 5% level.

The genes targeted by chi-miR-4110 were predicted by bioinformatics analytical tools. Results showed that chi-miR-4110 targeted the *Smad2* gene, with putative binding sites at positions 1706–1727 of the *Smad2* gene (XM_005697184.2; Fig 1A). To validate the identified direct binding site, luciferase activity was analysed by a luciferase reporter system. The wild-type psiCHECK-2–Smad2–3′UTR (WT) vector and mutant psiCHECK-2–Smad2–3'UTR (Mutant) vector were constructed (Fig 1B), with the latter containing six mutant nucleotides (Fig 1C). The WT and Mutant vectors were co-transfected with either chi-miR-4110 mimics or mimics NC. The WT group exhibited significantly inhibited luciferase activity (*P*< 0.05) compared with mimics NC and mutation groups (Fig 1D).

**Fig 2:**
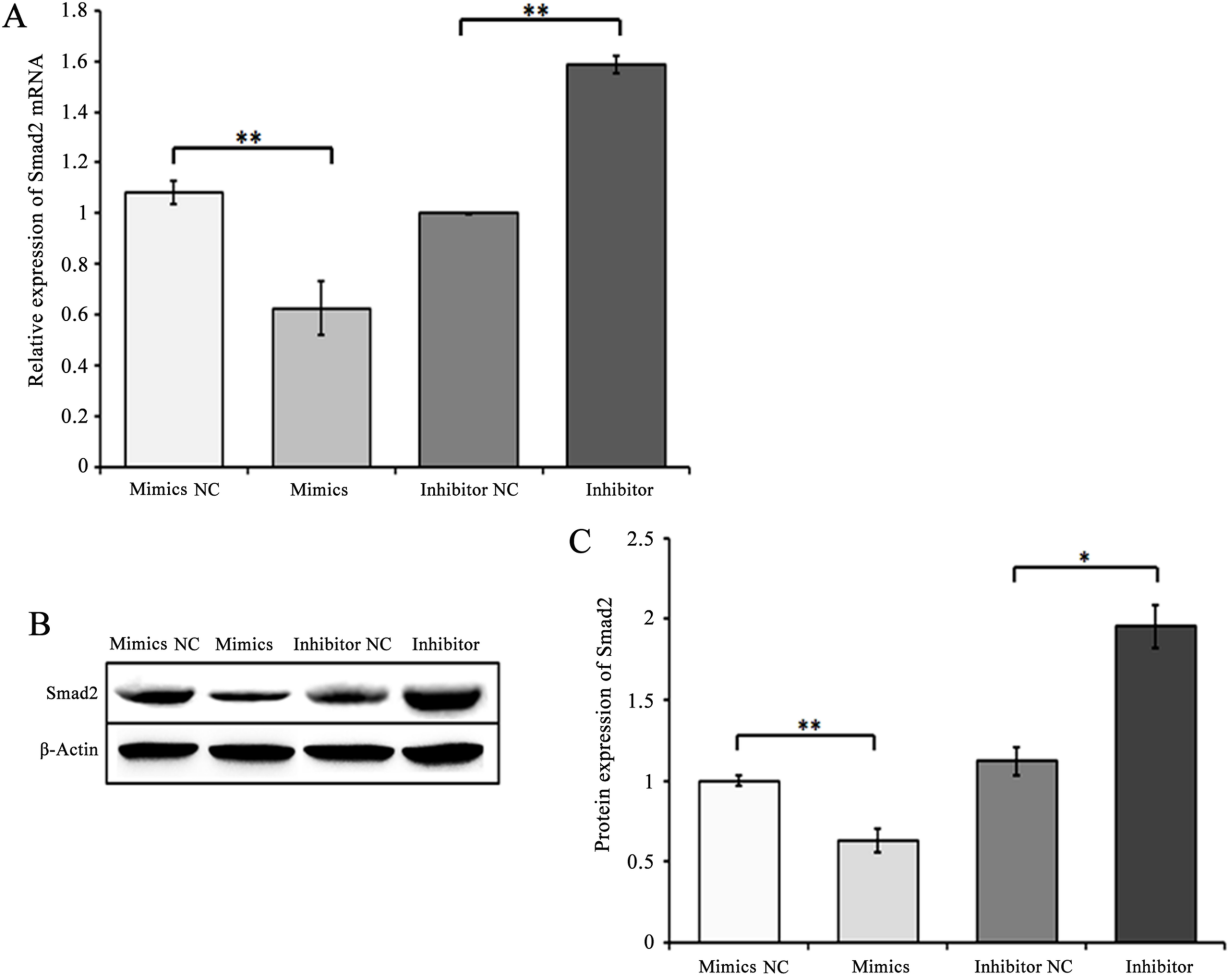
Increasing chi-miR-4110 level notably decreased Smad2 mRNA and protein levels. Real-time RCR (A) and western blot analysis (B and C) showed Smad2 mRNA and protein expression in GCs from goat ovaries cultured with mimics NC, chi-miR-4110 mimics (Mimics), chi-miR-4110 inhibitor (Inhibitor) or inhibitor NC. Data are presented as means ± standard deviation. *P*<0.05 is shown as *, and *P*<0.01 is shown as **.

To further determine whether chi-miR-4110 actually targeted the *Smad2* gene, goat GCs were transfected with mimics NC, chi-miR-4110 mimics, chi-miR-4110 inhibitor or inhibitor NC. The level of chi-miR-4110 in chi-miR-4110 mimics group was more than 680 times higher than that of endogenous chi-miR-4110 (*P*<0.01; S1 Fig). Real-time PCR and western blot analysis revealed that Smad2 mRNA and protein levels were significantly lower in GCs transfected with chi-miR-4110 mimics than in GCs transfected with mimics NC (*P*<0.01; Fig 2). In addition, Smad2 mRNA and protein levels were markedly higher in the chi-miR-4110 inhibitor group than in the inhibitor NC group (*P*< 0.05 or 0.01).

### Chi-miR-4110 regulates Sp1, FoxC1 and bHLHe22 expression by targeting Smad2

**Fig 3:**
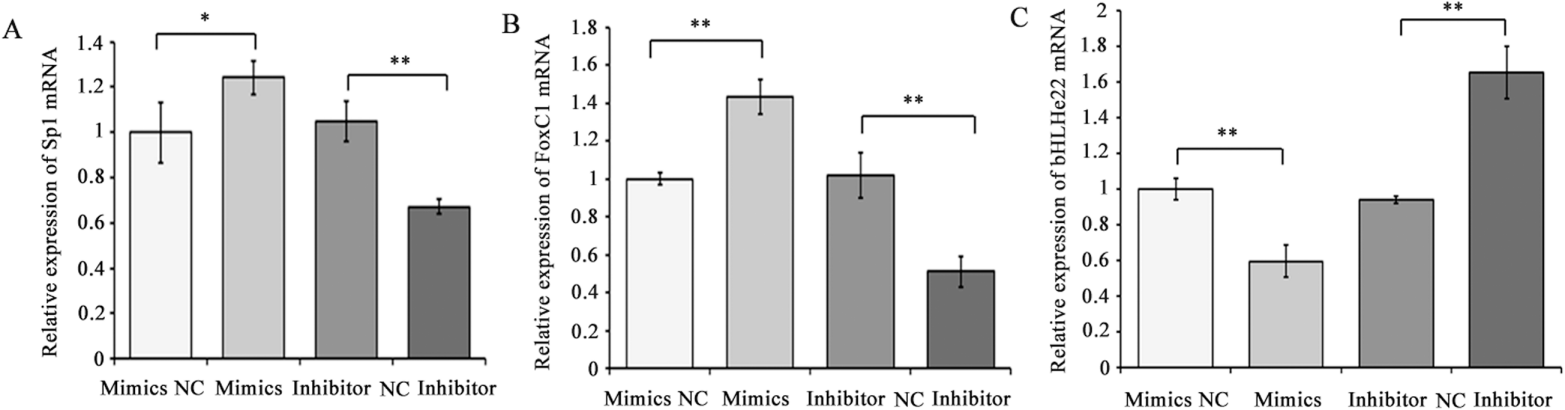
Chi-miR-4110 regulated Sp1, FoxC1 and bHLHe22 mRNA expression. (A) The effect of chi-miR-4110 on Sp1mRNA expression. (B) The effect of chi-miR-4110 on FoxC1 mRNA expression. (C) The effect of chi-miR-4110 on bHLHe22 mRNA expression. Sp1, FoxC1 and bHLHe22 mRNA expression levels were investigated in GCs transfected with chi-miR-4110 mimics, mimics NC, chi-miR-4110 inhibitor or inhibitor NC by real-time PCR. *GADPH* expression was used as a loading control. Data are presented as means ±standard deviation. *P*<0.05 is shown as *, and *P*<0.01 is shown as **.

**Fig 4:**
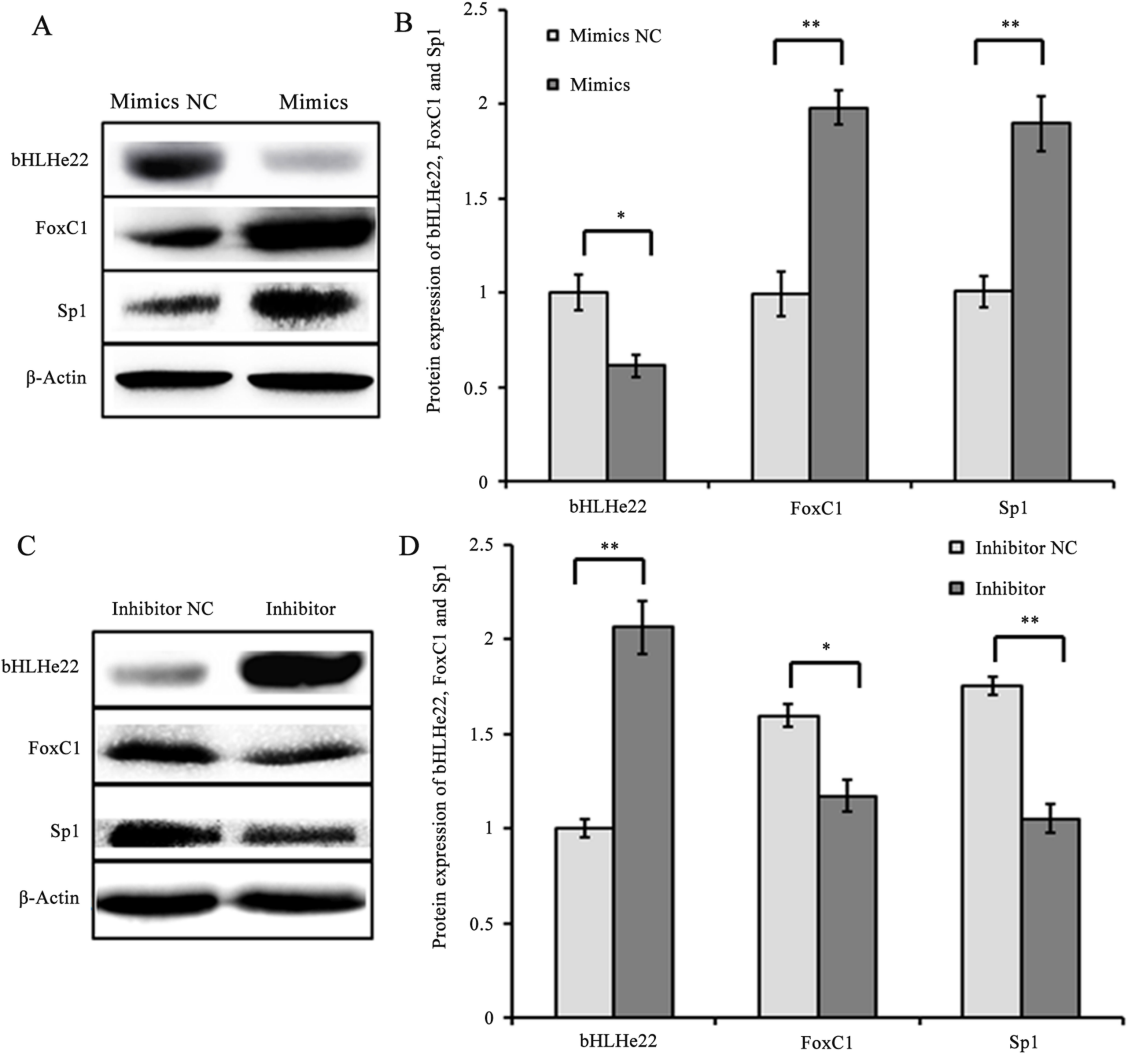
Effects of chi-miR-4110 on Sp1, FoxC1 and bHLHe22 protein levels. A and C: Western blot analysis results. B and D: Densitometric quantification of western blot results. Protein levels were normalised to β-Actin. Data are presented as means ± standard deviation. *P*<0.05 is shown as *, and *P*<0.01 is shown as **.

To explore the effects of chi-miR-4110 on Sp1, FoxC1 and bHLHe22 expression, the expression level of chi-miR-4110 was increased or decreased with chi-miR-4110 mimics or inhibitor in GCs. The mRNA and protein expression levels of FoxC1 and Sp1 markedly increased in the chi-miR-4110 mimics group compared with that of mimics NC group (*P*<0.01 or 0.05; Fig 3A and 3B and Fig 4A and 4B). In addition, bHLHe22 mRNA and protein levels significantly decreased in the chi-miR-4110 mimics group compared with that of mimics NC group (*P*<0.01 or 0.05; Fig 3C and Fig 4A and 4B). By contrast, FoxC1 and Sp1 mRNA and protein levels decreased in the chi-miR-4110 inhibitor group, whereas bHLHe22 mRNA and protein levels increased in the inhibitor NC group (*P*<0.01 or 0.05; Fig 3A–3C and Fig 4C and 4D).

**Fig 5:**
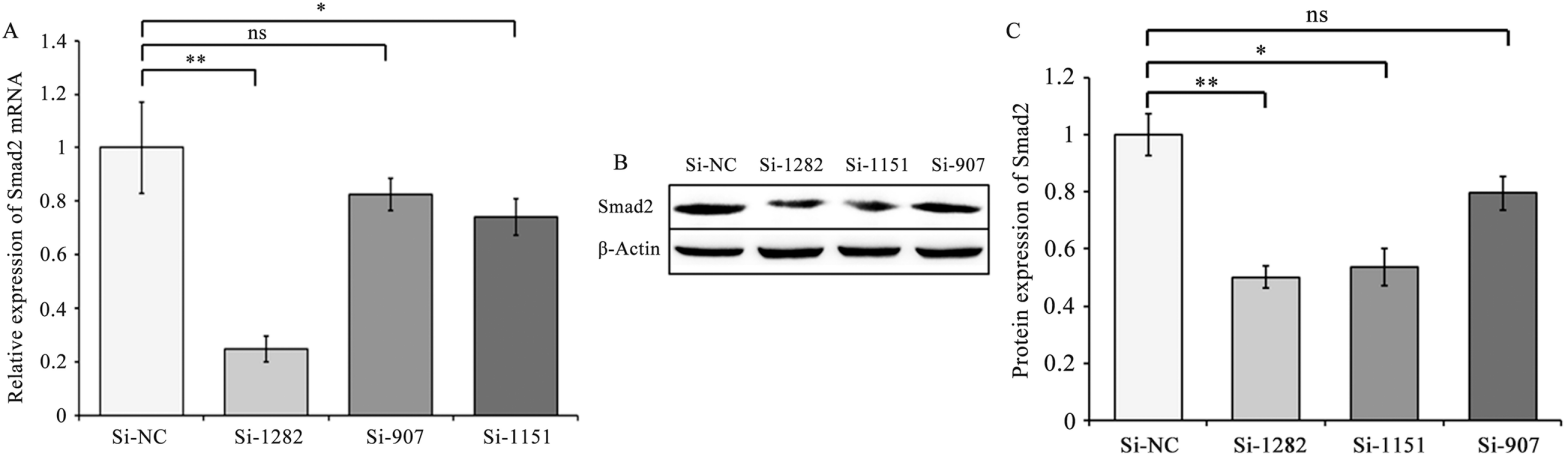
Smad2 siRNA treatment decreased Smad2 mRNA expression. Real-time PCR (A) and western blot analysis (B and C) showed Smad2 mRNA and protein expression in GCs from goat ovaries cultured with Si-NC, Si-907, Si-1151 and Si-1282. Data are presented as means ± standard deviation. *P*<0.05 is shown as *, and *P*<0.01 is shown as **. “ns” represents no significant difference.

**Fig 6:**
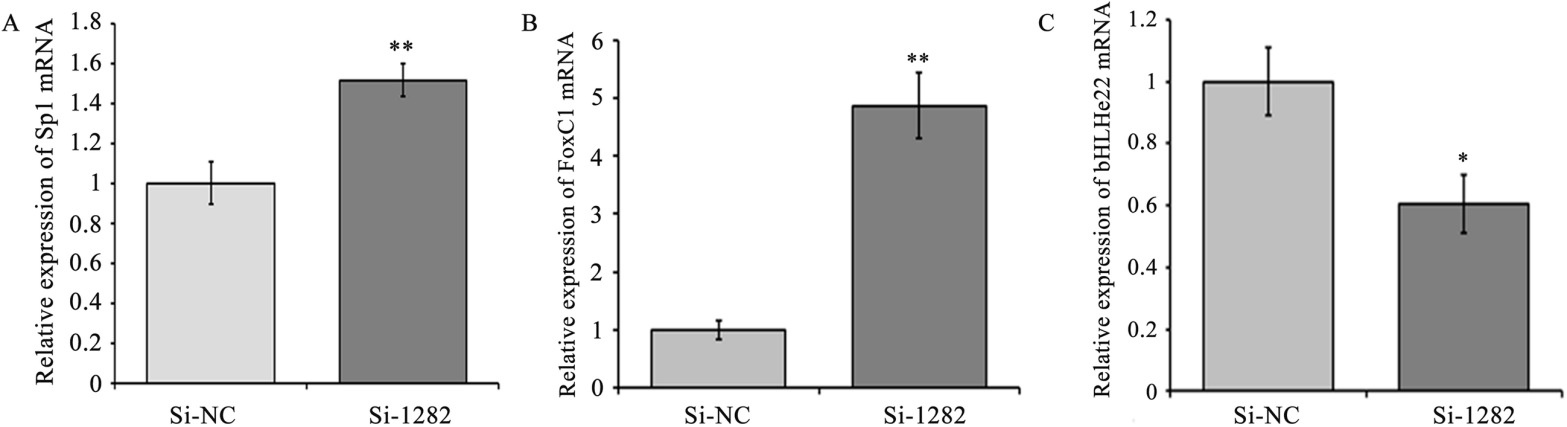
Si-1282 regulated Sp1, FoxC1 and bHLHe22 mRNA expression. (A) The effect of Si-1282 on Sp1 mRNA expression. (B) The effect of Si-1282 on FoxC1 mRNA expression. (C) The effect of Si-1282 on bHLHe22 mRNA expression. *GAPDH* expression was used as a loading control. Data are presented as means ± standard deviation. *P*<0.05 is shown as *, and *P*<0.01 is shown as **.

**Fig 7:**
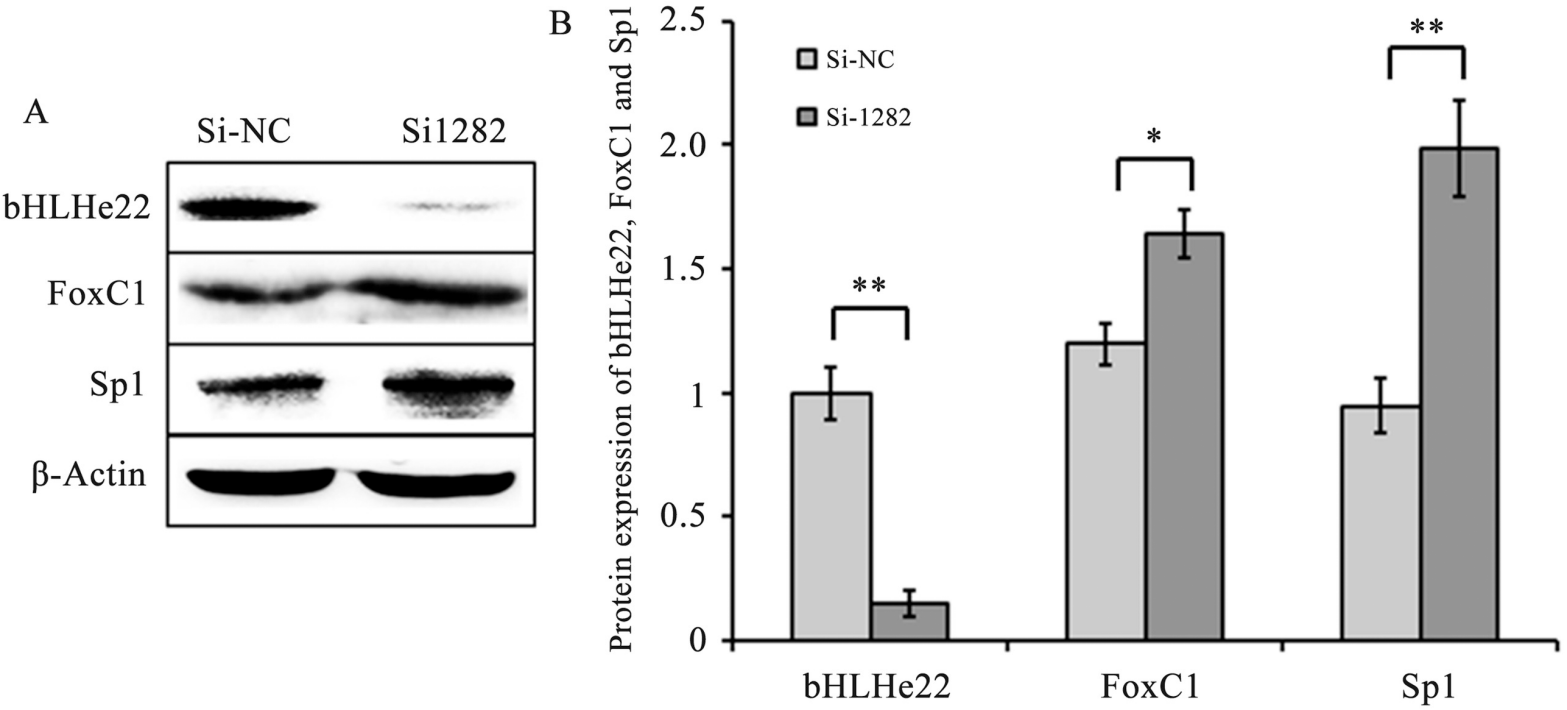
Effects of Si-1282 on Sp1, FoxC1 and bHLHe22 protein levels. (A) Western blot analysis results. (B) Densitometric quantification of western blot results. Protein levels were normalised to β-Actin. Data are presented as means ± standard deviation. *P*<0.05 is shown as *, and *P*<0.01 is shown as **.

To detect the effects of Smad2 on Sp1, FoxC1 and bHLHe22 expression, three siRNAs (Si-907, Si-1151and Si-1282) and Si-NC were transfected into GCs. Smad2 mRNA and protein levels significantly decreased in the Si-1151 and Si-1282 groups (*P*<0.01 or 0.05, Fig 5) compared with the Si-NC group. Given that Smad2 had the lowest expression level in the Si-1282 group, GCs were transfected with Si-1282 to confirm the effects of Smad2 on Sp1, FoxC1 and bHLHe22. The results showed that Sp1 and FoxC1 mRNA and protein levels were higher in the Si-1282 group than in the Si-NC group (*P*<0.01 or 0.05, Fig 6A and 6B and Fig 7A and 7B). In addition, bHLHe22 mRNA and protein levels were lower in the Si-1282 group than in the Si-NC group (*P*<0.01 or 0.05, Fig 6C and Fig 7A and 7B). Si-1282 interference results were consistent with experimental results of the chi-miR-4110 mimics, but contrary to those of the chi-miR-4110 inhibitor (Fig 3A–3C and Fig 4A–4D). These results suggested that chi-miR-4110 regulates Sp1, FoxC1 and bHLHe22 expression by targeting Smad2.

### Increasing chi-miR-4110 level promotes GC apoptosis

**Fig 8:**
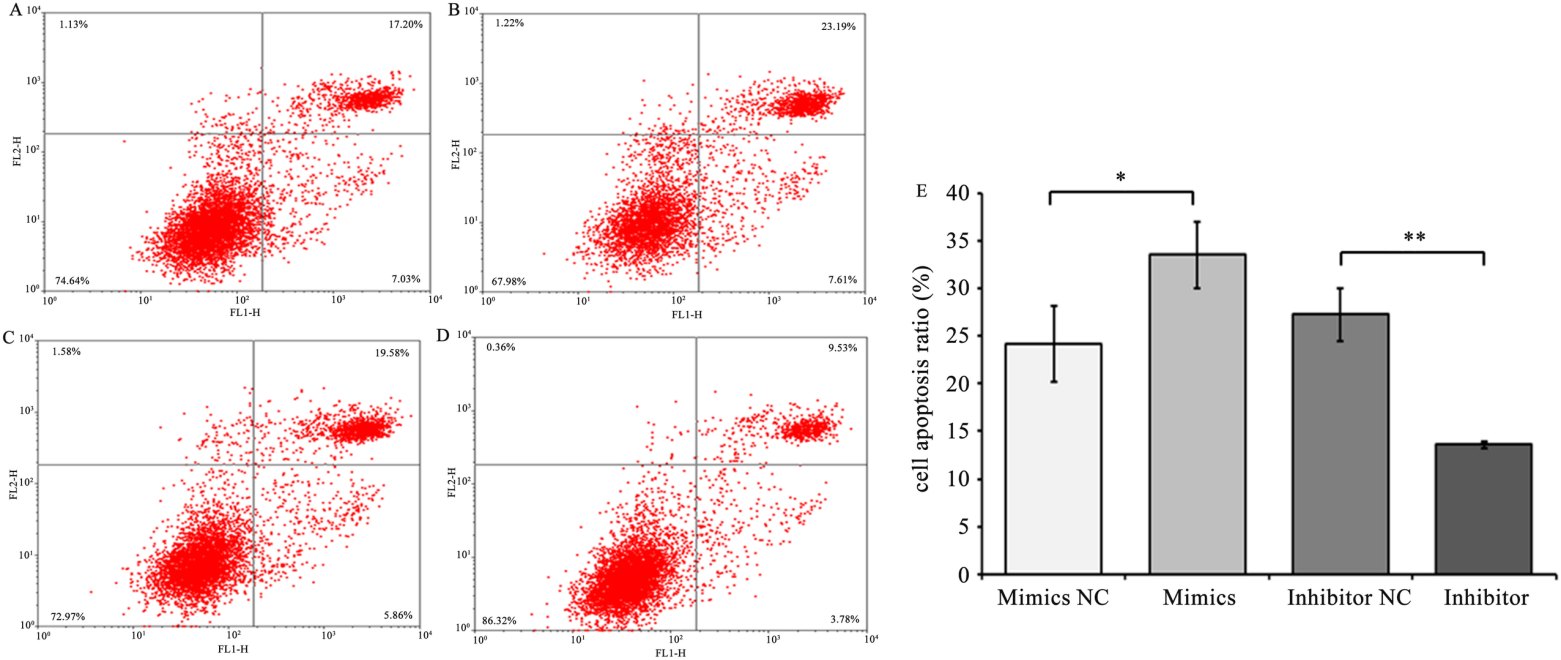
Chi-miR-4110 mediated GC apoptosis. (A) GCs transfected with mimics NC. (B) GCs transfected with chi-miR-4110 mimics. (C) GCs transfected with inhibitor NC. (D) GCs transfected with inhibitor. Cells were stained with Annexin V-FITC and PI. Apoptosis rate was determined by flow cytometry. (E) Apoptosis rate in mimics NC, chi-miR-4110 mimics, inhibitor NC and inhibitor. Data are presented as means ± standard deviation. *P*<0.05 is shown as *, and *P*<0.01 is shown as **.

**Fig 9:**
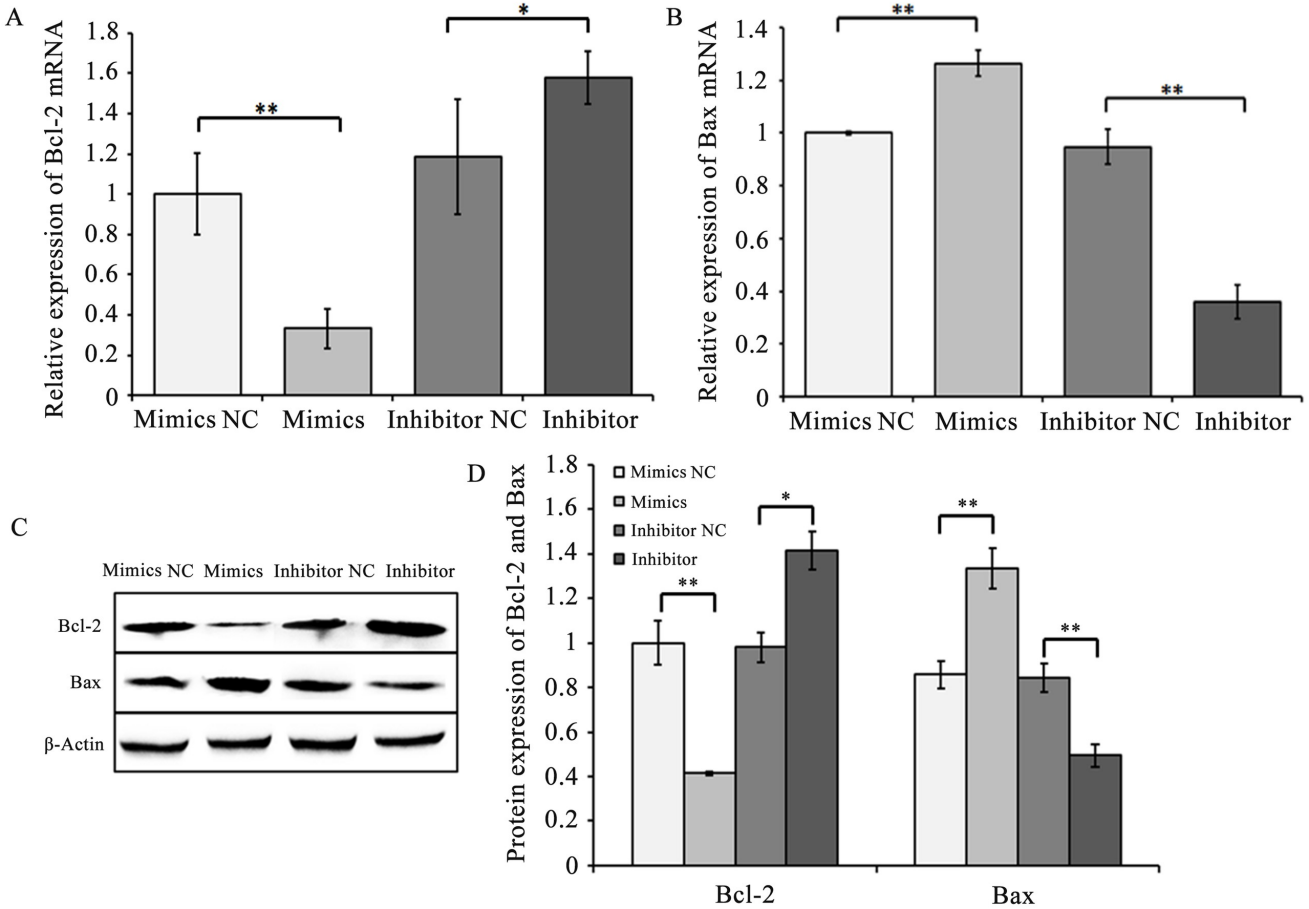
Chi-miR-4110 mimics and inhibitor affected Bc1-2 and Bax expression levels. (A and B) Bcl-2 and Bax mRNA levels in GCs transfected with mimics NC, chi-miR-4110 mimics, inhibitor NC and inhibitor were determined by real-time PCR. Relative gene expression was normalised to GAPDH. (C) Bcl-2 and Bax protein levels were determined by western blot and normalised to β-Actin. (D) Band densities were quantified with ImageJ software. The histogram presents the statistical results. Data are presented as means ± standard deviation. *P*<0.05 is shown as *, and *P*<0.01 is shown as **.

GC apoptosis is a key factor of goat follicular atresia. To confirm the effects of chi-miR-4110 on GC apoptosis *in vitro*, cultured GCs were transfected with chi-miR-4110 mimics and inhibitor. The apoptosis rate of GCs transfected with chi-miR-4110 mimics or inhibitor was examined by flow cytometry (Fig 8A–8D). The number of apoptotic cells was significantly higher in GCs transfected with chi-miR-4110 mimics (*P*<0.05) than in GCs transfected with mimics NC (Fig 8E). In addition, the number of apoptotic cells was lower inthechi-miR-4110 inhibitor group than in the inhibitor NC group (*P*<0.01). The relative expression levels of apoptosis-associated genes, including Bcl2-associated X protein (Bax) and B-cell lymphoma 2 (Bcl-2), were determined. Bax mRNA and protein expression levels (*P*<0.01) significantly increased, whereas BCL-2 expression levels (*P*<0.01) significantly decreased in GCs transfected with chi-miR-4110 mimics than in GCs transfected with mimics NC (Fig 9A–9D). The results of chi-miR-4110 inhibitor were contrary to those of the chi-miR-4110 mimics. Our results showed that increasing chi-miR-4110 level strongly promotes apoptosis.

### Smad2 knockdown in GCs increases apoptosis

**Fig 10:**
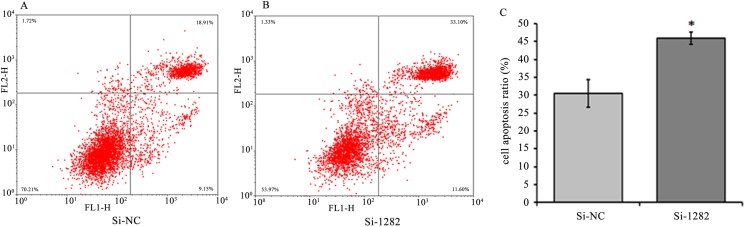
Si-1282 mediated GC apoptosis. (A) GCs transfected with Si-NC. (B) GCs transfected with Si-1282. Cells were stained with Annexin V-FITC and PI. Apoptosis rate was determined by flow cytometry. (C) Apoptosis rate in Si-NC and Si-1282. Data are presented as means ± standard deviation. *P*<0.05 is shown as *.

**Fig 11:**
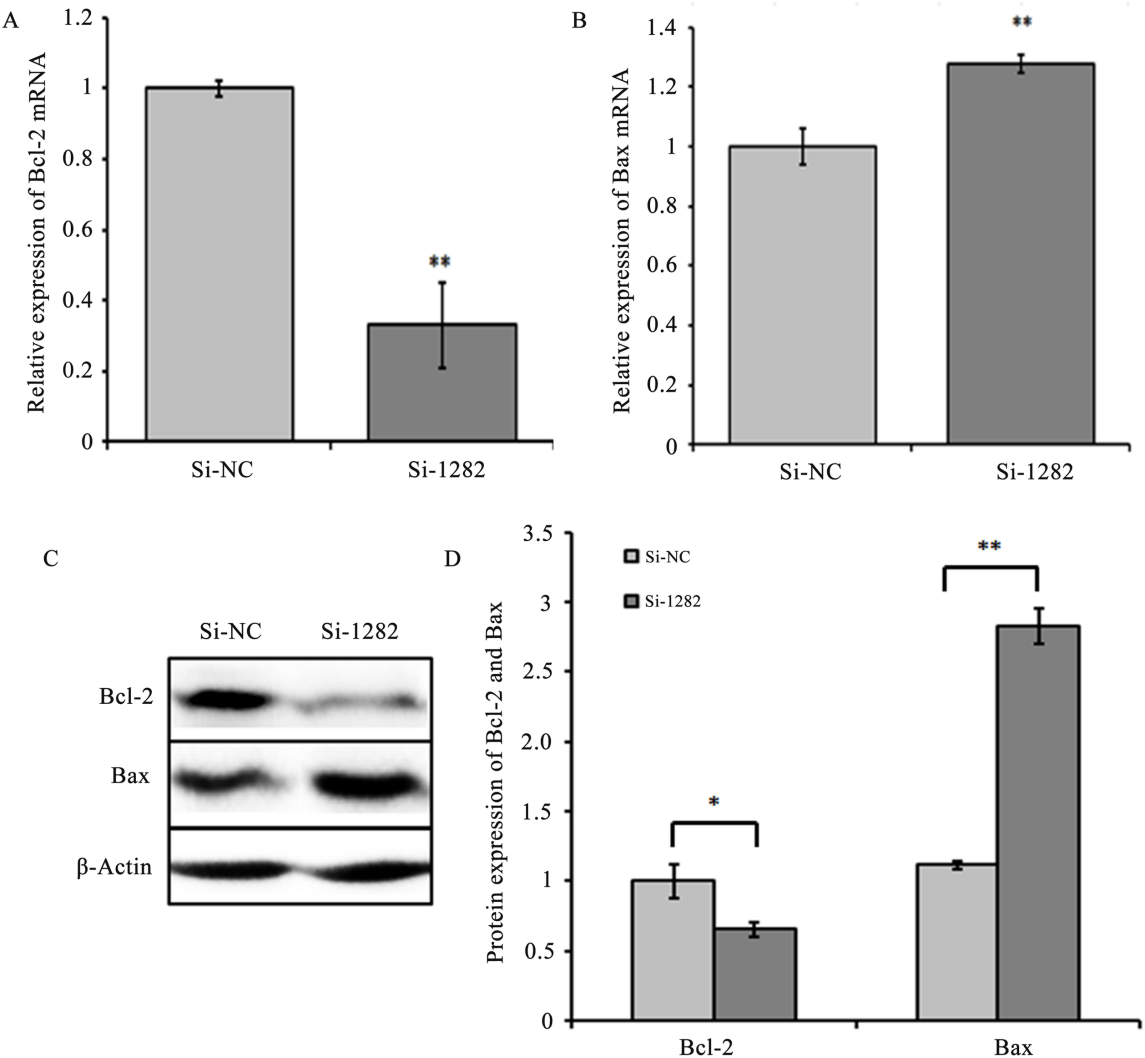
Si-1282 affected Bcl-2 and Bax expression levels. (A and B) Bcl-2 and Bax mRNA levels in GCs transfected with Si-NC and Si-1282 were determined by real-time PCR. Relative gene expression was normalised to *GAPDH*. (C) Bcl-2 and Bax protein levels were determined by western blot and normalised to β-Actin. (D) Band densities were quantified by ImageJ software. The histogram presents the statistical results. Data are presented as means ± standard deviation. *P*<0.05 is shown as *, and *P*<0.01 is shown as **.

To evaluate the effect of Smad2 on GC apoptosis *in vitro*, Smad2 expression in cultured GCs was knocked down by RNA interference (Si-1282). The apoptosis rate of Smad2-knockdown GCs was examined by flow cytometry (Fig 10A and 10B). The number of apoptotic cells was significantly higher in the Si-1282 group (*P*<0.05) than in the Si-NC-transfected GCs (Fig 10C), indicating that Smad2 loss-of-function is associated with apoptosis *in vitro*. Bax mRNA and protein expression levels significantly increased (*P*<0.01), whereas Bcl-2 expression levels (*P*<0.01 or 0.05) significantly decreased in GCs transfected with Si-1282 (Fig 11A–11D). The Si-1282 interference results were consistent with the experimental results of the chi-miR-4110 mimics, but contrary to those of the chi-miR-4110 inhibitor. These results suggested that chi-miR-4110 promotes GC apoptosis by Smad2.

## Discussion

Cell proliferation is critical in normal reproductive processes [24], including ovarian development, follicle progression from the primordial stage to the primary, secondary and tertiary or Graafian stages and ovulation [25]. However, because more than 99% of ovarian follicles undergo degeneration and atresia, less than 1% of the ovarian follicles reach the ovulation stage in mammals [26]. Previous studies demonstrated that GC apoptosis triggers follicular atresia [27], but the molecular mechanisms involved in this process are still unclear. In domestic animals, promoting follicular development, maturation and ovulation, whilst inhibiting GC apoptosis and follicular atresia may improve animal fertility, specifically ovulation and litter size. Therefore, GC apoptosis and follicular atresia analyses have become hotspots in animal science research [28–30]. Zhou et al. (2015) demonstrated that let-7g regulates GCs apoptosis in pig ovaries by targeting TGFBR1 and down regulating the TGF-β signalling pathway [20]. MiR-320 regulates GC proliferation and the production of testosterone, progesterone and estradiol by targeting E2F1 and SF-1 during follicular development [25]. Grossman et al. (2015) demonstrated that human chorionic gonadotropin (hCG) decreases miR-125a-3p expression towards ovulation, enabling mural GC migration. This process is partly mediated by Fyn and may be important for proper ovulation [31]. In this study, we demonstrated that chi-miR-4110 promotes GC apoptosis by targeting Smad2 in the caprine ovary.

**Fig 12:**
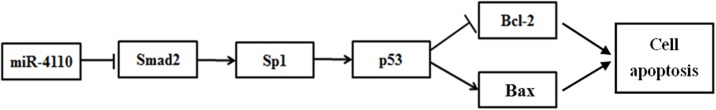
Proposed model of chi-miR-4110 regulation in caprine GCs. Chi-miR-4110 represses Smad2, increasing Sp1 expression; the increaseinSp1 induces p53-upregulated modulator of apoptosis (PUMA), which increases the relative abundance of Bax and causes caprine GC apoptosis.

Predicting miRNA target genes by computational analysis is efficient, but can result in tens or hundreds of target genes with high false-positive rates [32]. To avoid this issue, this study executed a luciferase assay to identify a chi-miR-4110binding site in the 3′UTR of *Smad2* mRNA, confirming that the *Smad2* mRNA 3′UTR from 1706bp to 1727bp is the chi-miR-4110 binding site. Initial reports posited that the extent of complementarity between miRNA and its target mRNA governs either translational mRNA cleavage or repression [33]. In plants, nearly perfect complementarity leads to the degradation of target miRNAs [34], whereas in animals, partial complementarity results in a translational block [35]. However, recent studies have reported that miRNAs can also induce mRNA degradation in animals and, conversely, translational repression in plants [36]. Furthermore, the state of the complementarity between target mRNA and miRNA possibly affects mRNA degradation [37, 38]. In this study, because the chi-miR-4110 inhibitor has a sequence reversed with chi-miR-4110, and binds specifically to the endogenous chi-miR-4110 in the cells to knock out the endogenous chi-miR-4110, attenuate its effect on target gene silencing and enhance target protein expression, the Smad2 mRNA and protein levels of the chi-miR-4110 inhibitor group were markedly higher than those of the inhibitor NC group. In addition, the results presented in this study demonstrated that chi-miR-4110 decreased Smad2 protein production by altering *Smad2* mRNA levels. It is arguable whether Smad2 protein reduction by chi-miR-4110 is significant for physiological functions in the ovary. Previous reports implicated Activin, which signals through Smad2 and Smad3, in the regulation of early oocyte development [39]. Treating newborn pups with Activin increases the survival of germ cells and assembly into primordial follicles [40]. In the canonical pathway, the downstream signalling molecules, Smad2 and Smad3, are phosphorylated and activated when the ligand binds to the receptor. In combination with common Smad4, they subsequently translocate into the nucleus where these molecules mediate TGF-β1-regulated gene expression in GCs [41]. In this study, chi-miR-4110 repressed Smad2, increasing Sp1 expression. The increase in Sp1 induces p53-upregulated modulator of apoptosis (PUMA), which is essential to p53-dependent apoptosis following DNA damage (Fig 12), subsequently increasing the relative abundance of Bax and causing caprine GC apoptosis [42].

## Conclusions

We demonstrate that chi-miR-4110 promoted GC apoptosis in the caprine ovary by directly targeting and down regulating the *Smad2* gene, showing therapeutic implications in the control of fertility. We note that our results do not exclude the possibility that other ovarian functions, such as folliculogenesis, oocyte maturation and ovulation, are regulated by chi-miR-4110. However, our results show that ovarian GC apoptosis is sensitive to chi-miR-4110. Although the precise mechanism regulating GC apoptosis is not elucidated, our findings may provide relevant data for those investigating miRNA-mediated regulation of ovarian functions.

## Supporting information

S1 Fig Concentration analysis of endogenous chi-miR-4110 and chi-miR-4110 mimics. (TIF)

## References

[1] Gebremedhn S, Salilew-Wondim D, Ahmad I, Sahadevan S, Hossain MM, Hoelker M, et al MicroRNA expression profile in bovine granulosa cells of preovulatory dominant and subordinate follicles during the late follicular phase of the estrous cycle. Plos One. 2015; 10(5):e0125912 doi: 10.1371/journal.pone.0125912 25993098 10.1371/journal.pone.0125912 PMC4438052

[2] Bradford AP, Jones K, Kechris K, Chosich J, Montague M, Warren WC, et al Joint MiRNA/mRNA expression profiling reveals changes consistent with development of dysfunctional corpus luteum after weight gain. Plos One. 2015; 10(8):e0135163 doi: 10.1371/journal.pone.0135163 26258540 10.1371/journal.pone.0135163 PMC4530955

[3] Knight PG, Glister C. TGF-beta superfamily members and ovarian follicle development. Reproduction. 2006; 132(2):191–206. doi: 10.1530/rep.1.01074 . 16885529 10.1530/rep.1.01074

[4] Fenwick MA, Mora JM, Mansour YT, Baithun C, Franks S, Hardy K. Investigations of TGF-beta signaling in preantral follicles of female mice reveal differential roles for bone morphogenetic protein 15. Endocrinology. 2013; 154(9):3423–36. doi: 10.1210/en.2012-2251 . 23782946 10.1210/en.2012-2251

[5] Liu L, Liu X, Ren X, Tian Y, Chen Z, Xu X, et al Smad2 and Smad3 have differential sensitivity in relaying TGFβ signaling and inversely regulate early lineage specification. Scientific Reports. 2016; 6:21602 doi: 10.1038/srep21602 26905010 10.1038/srep21602 PMC4764856

[6] Liu J, Du X, Zhou J, Pan Z, Liu H, Li Q. MicroRNA-26b functions as a proapoptotic factor in porcine follicular Granulosa cells by targeting Sma-and Mad-related protein 4. Biology of Reproduction. 2014; 91(6):146 doi: 10.1095/biolreprod.114.122788 25395673 10.1095/biolreprod.114.122788

[7] Tian X, Halfhill AN, Diaz FJ. Localization of phosphorylated SMAD proteins in granulosa cells, oocytes and oviduct of female mice. Gene Expression Patterns. 2010; 10(2–3):105–12. doi: 10.1016/j.gep.2010.02.003 20176141 10.1016/j.gep.2010.02.003

[8] Kaivo-oja N, Jeffery LA, Ritvos O, Mottershead DG. Smad signalling in the ovary. Reproductive Biology and Endocrinology. 2006; 4:21 doi: 10.1186/1477-7827-4-21 16611366 10.1186/1477-7827-4-21 PMC1459162

[9] Xing N, Liang YJ, Gao ZZ, He JP, He XY, Li HQ, et al Expression and localization of Smad2 and Smad4 proteins in the porcine ovary. Acta Histochem. 2014; 116(8):1301–6. doi: 10.1016/j.acthis.2014.07.014 25190106 10.1016/j.acthis.2014.07.014

[10] Attisano L, Wrana JL. Signal transduction by the TGF-beta superfamily. Science. 2002; 296(5573):1646–7. doi: 10.1126/science.1071809 12040180 10.1126/science.1071809

[11] Miyazawa K, Shinozaki M, Hara T, Furuya T, Miyazono K. Two major Smad pathways in TGF-beta superfamily signalling. Genes Cells. 2002; 7(12):1191–204. doi: 10.1046/j.1365-2443.2002.00599.x 12485160 10.1046/j.1365-2443.2002.00599.x

[12] Zhao L, He JP, Guo QY, Wen XX, Zhang XJ, Dong CS. Expression of growth differentiation factor 9 (GDF9) and its receptor in adult cat testis. Acta Histochem. 2011;113(8):771–6. doi: 10.1016/j.acthis.2010.11.005 21146857 10.1016/j.acthis.2010.11.005

[13] Li JX, Du SH, Sheng XJ, Liu J, Cen BH, Huang F, et al MicroRNA-29b inhibits endometrial fibrosis by regulating the Sp1-TGF-beta 1/Smad-CTGF axis in a rat model. Reproductive Sciences. 2016; 23(3):386–94. doi: 10.1177/1933719115602768 26392347 10.1177/1933719115602768

[14] Hopkins A, Mirzayans F, Berry F. Foxc1 expression in early osteogenic differentiation is regulated by BMP4-SMAD activity. Journal of Cellular Biochemistry. 2016; 117(7):1707–17. doi: 10.1002/jcb.25464 26666591 10.1002/jcb.25464

[15] Ambros V. The functions of animal microRNAs. Nature. 2004; 431(7006):350–5. doi: 10.1038/nature02871 15372042 10.1038/nature02871

[16] Christenson LK. MicroRNA control of ovarian function. Animal Reproduction. 2010; 7(3):129–33. 21666774 PMC3111027

[17] Perdas E, Stawski R, Nowak D, Zubrzycka M. The role of miRNA in papillary thyroid cancer in the context of miRNA Let-7 family. International Journal of Molecular Sciences. 2016; 17(6). doi: 10.3390/ijms17060909 27314338 10.3390/ijms17060909 PMC4926443

[18] Iwamune M, Nakamura K, Kitahara Y, Minegishi T. MicroRNA-376a regulates 78-kilodalton glucose-regulated protein expression in rat granulosa cells. Plos One. 2014; 9(10):e108997 doi: 10.1371/journal.pone.0108997 25279841 10.1371/journal.pone.0108997 PMC4184830

[19] Maalouf SW, Liu WS, Pate JL. MicroRNA in ovarian function. Cell and Tissue Research. 2016; 363(1):7–18. doi: 10.1007/s00441-015-2307-4 26558383 10.1007/s00441-015-2307-4

[20] Zhou J, Liu J, Pan Z, Du X, Li X, Ma B, et al The let-7g microRNA promotes follicular granulosa cell apoptosis by targeting transforming growth factor-beta type 1 receptor. Molecular and Cellular Endocrinology. 2015; 409:103–12. doi: 10.1016/j.mce.2015.03.012 . 25817543 10.1016/j.mce.2015.03.012

[21] An X, Song Y, Hou J, Li G, Zhao H, Wang J, et al Identification and profiling of microRNAs in the ovaries of polytocous and monotocous goats during estrus. Theriogenology. 2016; 85(4):769–80. doi: 10.1016/j.theriogenology.2015.09.056 . 26542138 10.1016/j.theriogenology.2015.09.056

[22] Peng JY, Gao KX, Xin HY, Han P, Zhu GQ, Cao BY. Molecular cloning, expression analysis, and function of decorin in goat ovarian granulosa cells. Domestic Animal Endocrinology. 2016; 57:108–16. doi: 10.1016/j.domaniend.2016.05.006 27565237 10.1016/j.domaniend.2016.05.006

[23] Peng J, Xin H, Peng H, Gao K, Gao T, Lei Y, et al Expression and regulative function of tissue inhibitor of metalloproteinase 3 in the goat ovary and its role in cultured granulosa cells. Molecular and Cellular Endocrinology. 2015; 412(26):104–15. doi: 10.1016/j.mce.2015.06.001 26054746 10.1016/j.mce.2015.06.001

[24] Stouffer RL, Xu F, Duffy DM. Molecular control of ovulation and luteinization in the primate follicle. Frontiers in Bioscience. 2007; 12:297–307. 17127300 10.2741/2065

[25] Yin M, Wang X, Yao G, Lu M, Liang M, Sun Y, et al Transactivation of micrornA-320 by microRNA-383 regulates granulosa cell functions by targeting E2F1 and SF-1 proteins. Journal of Biological Chemistry. 2014; 289(26):18239–57. doi: 10.1074/jbc.M113.546044 24828505 10.1074/jbc.M113.546044 PMC4140302

[26] Maeda A, Inoue N, Matsuda-Minehata F, Goto Y, Cheng Y, Manabe N. The role of interleukin-6 in the regulation of granulosa cell apoptosis during follicular atresia in pig ovaries. Journal of Reproduction and Development. 2007; 53(3):481–90. 17272928 10.1262/jrd.18149

[27] Matsuda F, Inoue N, Manabe N, Ohkura S. Follicular growth and atresia in mammalian ovaries: regulation by survival and death of granulosa cells. Journal of Reproduction and Development. 2012; 58(1):44–50 22450284 10.1262/jrd.2011-012

[28] Troppmann B, Kossack N, Nordhoff V, Schüring AN, Gromoll J. microRNA miR-513a-3p acts as a co-regulator of luteinizing hormone/chorionic gonadotropin receptor gene expression in human granulosa cells. Molecular and Cellular Endocrinology. 2014; 390(1–2):65–72. doi: 10.1016/j.mce.2014.04.003 24747085 10.1016/j.mce.2014.04.003

[29] Toms D, Xu S, Bo P, Wu D, Li J. Progesterone receptor expression in granulosa cells is suppressed by microRNA-378-3p. Molecular and Cellular Endocrinology. 2015; 399:95–102. doi: 10.1016/j.mce.2014.07.022 25150622 10.1016/j.mce.2014.07.022

[30] Gebremedhn S, Salilewwondim D, Hoelker M, Rings F, Neuhoff C, Tholen E, et al MicroRNA-183-96-182 cluster regulates bovine granulosa cell proliferation and cell cycle transition by coordinately targeting FOXO11. Biology of Reproduction. 2016; 94(6):127 doi: 10.1095/biolreprod.115.137539 27122636 10.1095/biolreprod.115.137539 PMC6702798

[31] Grossman H, Chuderland D, Ninio-Many L, Hasky N, Kaplan-Kraicer R, Shalgi R. A novel regulatory pathway in granulosa cells, the LH/human chorionic gonadotropin-microRNA-125a-3p-Fyn pathway, is required for ovulation. Faseb Journal. 2015; 29(8):3206–16. doi: 10.1096/fj.14-269449 25921829 10.1096/fj.14-269449

[32] Seitz H. Redefining MicroRNA Targets. Current Biology. 2009; 19(10): 870–3. doi: 10.1016/j.cub.2009.03.059 19375315 10.1016/j.cub.2009.03.059

[33] Bartel DP. MicroRNAs: genomics, biogenesis, mechanism, and function. Cell. 2004; 116(2):281–97. 14744438 10.1016/s0092-8674(04)00045-5

[34] Palatnik JF, Allen E, Wu X, Schommer C, Schwab R, Carrington JC, et al Control of leaf morphogenesis by microRNAs. Nature. 2003; 425(6955):257–63. doi: 10.1038/nature01958 12931144 10.1038/nature01958

[35] Chen XM. A microRNA as a translational repressor of APETALA2 in Arabidopsis flower development. Science. 2004;303(5666):2022–5. doi: 10.1126/science.1088060 12893888 10.1126/science.1088060 PMC5127708

[36] Carthew RW, Sontheimer EJ. Origins and Mechanisms of miRNAs and siRNAs. Cell. 2009; 136(4):642–55. doi: 10.1016/j.cell.2009.01.035 19239886 10.1016/j.cell.2009.01.035 PMC2675692

[37] Ameres SL, Horwich MD, Hung JH, Xu J, Ghildiyal M, Weng ZP, et al Target RNA-directed trimming and tailing of small silencing RNAs. Science. 2010; 328(5985):1534–9. doi: 10.1126/science.1187058 20558712 10.1126/science.1187058 PMC2902985

[38] Jiang L, Huang J, Li L, Chen Y, Chen X, Zhao X, et al MicroRNA-93 promotes ovarian granulosa cells proliferation through targeting CDKN1A in polycystic ovarian syndrome. Journal of Clinical Endocrinology and Metabolism. 2015; 100(5):E729–38. doi: 10.1210/jc.2014-3827 25695884 10.1210/jc.2014-3827 PMC4422895

[39] Tian X, Halfhill AN, Diaz FJ. Localization of phosphorylated SMAD proteins in granulosa cells, oocytes and oviduct of female mice. Gene Expression Patterns. 2010; 10(2–3):105–12. doi: 10.1016/j.gep.2010.02.003 20176141 10.1016/j.gep.2010.02.003

[40] Bristol-Gould SK, Kreeger PK, Selkirk CG, Kilen SM, Cook RW, Kipp JL, et al Postnatal regulation of germ cells by activin: The establishment of the initial follicle pool. Developmental Biology. 2006; 298(1):132–48. doi: 10.1016/j.ydbio.2006.06.025 16930587 10.1016/j.ydbio.2006.06.025

[41] Cheng JC, Chang HM, Fang L, Sun YP, Leung PCK. TGF-β1 up-pegulates connective tissue growth factor expression in human granulosa cells through Smad and ERK1/2 signaling pathways. Plos One. 2014; 10(5):e0126532 doi: 10.1371/journal.pone.0126532 25955392 10.1371/journal.pone.0126532 PMC4425519

[42] Ming L, Sakaida T, Yue W, Jha A, Zhang L, Yu J. Sp1 and p73 activate PUMA following serum starvation. Carcinogenesis. 2008; 29(10):1878–84. doi: 10.1093/carcin/bgn150 18579560 10.1093/carcin/bgn150 PMC2722853

