# Supplementary figures and images for: Chi-miR-4110 promotes granulosa cell apoptosis by targeting Sma- and Mad-related protein 2 (Smad2) in the caprine ovary
Source: PLoS One. 2017 Jul 13;12(7):e0181162. doi: 10.1371/journal.pone.0181162 (PMC5509297; doi:10.1371/journal.pone.0181162)

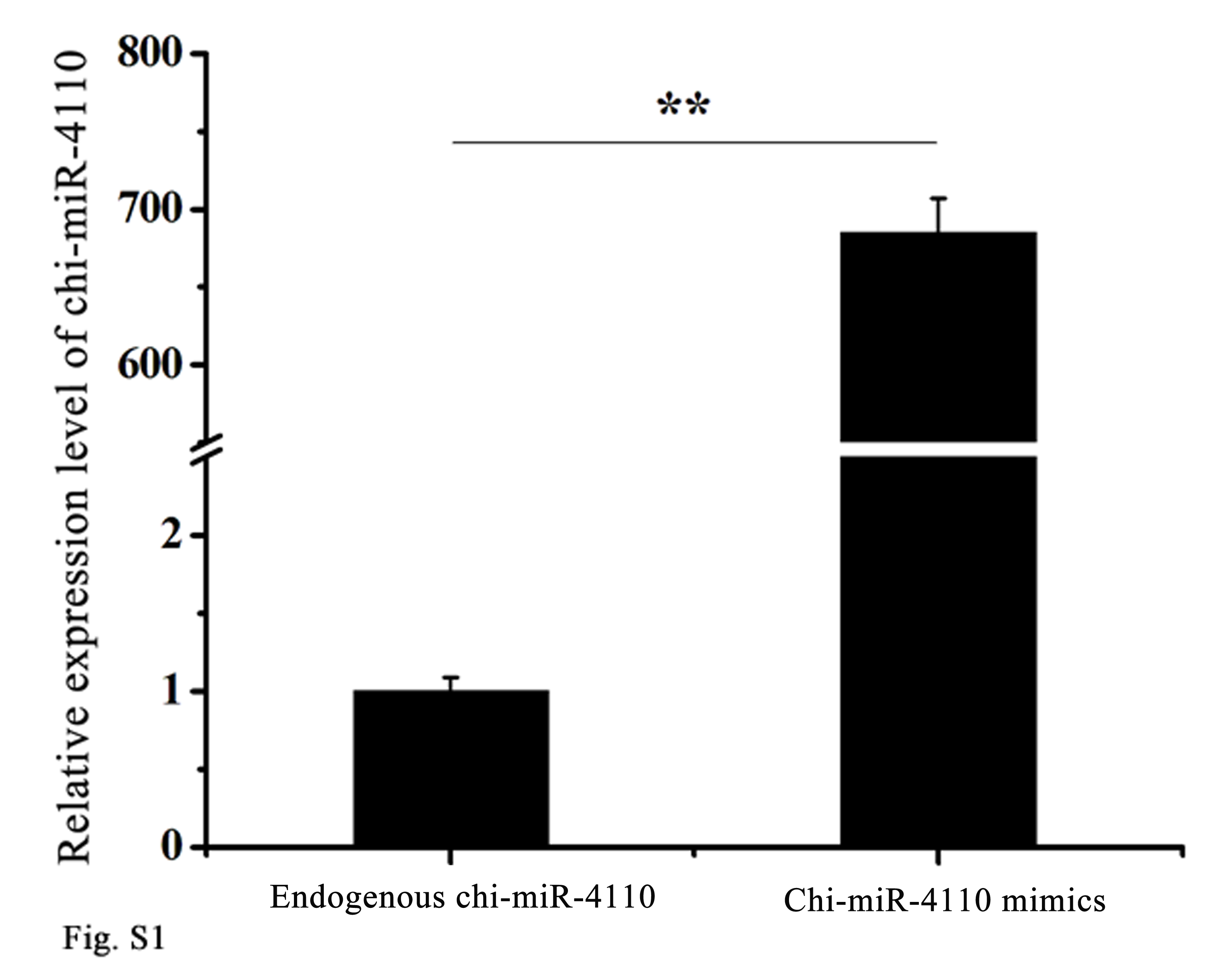

Supplement: S1 Fig — (TIF) [file pone.0181162.s001.tif]
